# Transfer of Cadmium from Soil to Vegetable in the Pearl River Delta area, South China

**DOI:** 10.1371/journal.pone.0108572

**Published:** 2014-09-23

**Authors:** Huihua Zhang, Junjian Chen, Li Zhu, Guoyi Yang, Dingqiang Li

**Affiliations:** 1 Guangdong Institute of Eco-environmental and Soil Sciences, Guangzhou, China; 2 Management School, Jinan University, Guangzhou, China; University of Vigo, Spain

## Abstract

The purpose of this study was to investigate the regional Cadmium (Cd) concentration levels in soils and in leaf vegetables across the Pearl River Delta (PRD) area; and reveal the transfer characteristics of Cadmium (Cd) from soils to leaf vegetable species on a regional scale. 170 paired vegetables and corresponding surface soil samples in the study area were collected for calculating the transfer factors of Cadmium (Cd) from soils to vegetables. This investigation revealed that in the study area Cd concentration in soils was lower (mean value 0.158 mg kg^−1^) compared with other countries or regions. The Cd-contaminated areas are mainly located in west areas of the Pearl River Delta. Cd concentrations in all vegetables were lower than the national standard of Safe vegetables (0.2 mg kg^−1^). 88% of vegetable samples met the standard of No-Polluted vegetables (0.05 mg kg^−1^). The Cd concentration in vegetables was mainly influenced by the interactions of total Cd concentration in soils, soil pH and vegetable species. The fit lines of soil-to-plant transfer factors and total Cd concentration in soils for various vegetable species were best described by the exponential equation (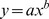), and these fit lines can be divided into two parts, including the sharply decrease part with a large error range, and the slowly decrease part with a low error range, according to the gradual increasing of total Cd concentrations in soils.

## Introduction

Cadmium is non-essential element to biota. It is known to be toxic for plants as well as animals to a much higher extent and at lower concentrations than e.g. Zn, Pb, or Cu. It especially affects humans because of their longevity and the accumulation of Cd in their organs by eating Cd-contaminated food [1]–[3].

Soil Cd is naturally derived from parent materials because of chemical weathering, and as a contaminant in many areas of anthropogenic activities such as mining, smelting, composts, phosphate fertilizer application, waste disposal, and vehicle exhausts [4]–[7]. The main forms of cadmium in soils and sediments are the exchangeable fraction, followed by the Fe-Mn oxides and residual fractions. Several studies indicated the Cd in soils contaminated by anthropogenic activates such as mining and smelting, seem to be more bioavailable than Cd from unimpacted soils [5]. To protect the safe of soil environment and prevent the soil Cd contamination, various soil quality standards were established in many countries or regions, such as 0.3 mg kg^−1^ in China [8], 0.8 mg kg^−1^ in the Netherlands and Switzerland, 0.5–1 mg kg^−1^ in Austria [9], and 5 mg kg^−1^ in Taiwan [10].

Because soil Cd is easily accumulated by plants, soil-plant-human transfer of Cd has been considered as a major pathway of human exposure to soil Cd [11], [12]. Many studies have been conducted in China such as Cd in rice [10], [13]–[15], in orchard [16], [17], and in vegetable [18]–[21]. And the maximum permissible concentrations of Cd of 0.05 mg kg^−1^ for non-environmental pollution vegetable – so called “No-Polluted vegetable” [22] and maximium concentration of 0.2 mg kg^−1^ in leaf vegetables for food security – so called “Safe vegetable” [23] are used in China.

Although undoubtedly soil Cd is the primary source of vegetable Cd, and total soil Cd concentration was commonly used for soil environmental quality estimation, its usefulness to predict soil-to-plant transfer was often questioned since the phytoavailability of Cd in soils [12], [21], [24]. Yang et al [25] proposed that various vegetable species showed significantly different accumulation capacity for Cd in the same soil sample sites; Soil-to-vegetable transfer factor (TF) of Cd was much higher in a pot experiment than that in a field trial. Previous studies have showed that the TF is decided especially by the soil properties and plant species [26]. Wang et al [12] pointed out that TFs can be considered as a useful index of metals potentially transfer abilities from soil to plant, and the TFs of Cd for leafy vegetables were higher than those for non-leafy vegetables.TF has been widely used in the evaluation of potential health risk of human exposure to metals from soil [3], [5], [12], [15], [16].

In the PRD area, the vegetable planting area is about 4830 km^2^. The vegetable production was 1129.2×10^4^ tons in 2011. The leaf vegetable is the dominant consumption for Guangdong people, the planting area is about 33% of the total planting area [27]. As a report of the Statistics Bureau of Guangdong Province, the export of fresh vegetable was up to 78.3 ×10^4^ tons [28], mainly selled to Japan, Korea, Malaysia, Russia, and America. Hence, the quality safety of vegetable is not only concerned by the local government, but it should receive much more attention from the international community for the increasing export and import trade.

In this study, we conducted a systemic environmental quality survey of the vegetable soil and various vegetables in the PRD area. The aim of this study was to: (1) obtain the information on concentrations and spatial patterns of Cd in soils and leaf vegetables across the PRD area; (2) investigate the relationship between soil Cd and vegetable Cd on a regional scale; (3) reveal the transfer characteristic of Cd from soil to various leaf vegetable species.

## Materials and Methods

### Ethics Statement

This study was carried out on collective-owned lands, and the owners of the lands gave us permission to conduct the study on these sites. The field studies did not involve endangered or protected species.

### Study area

**Figure 1:**
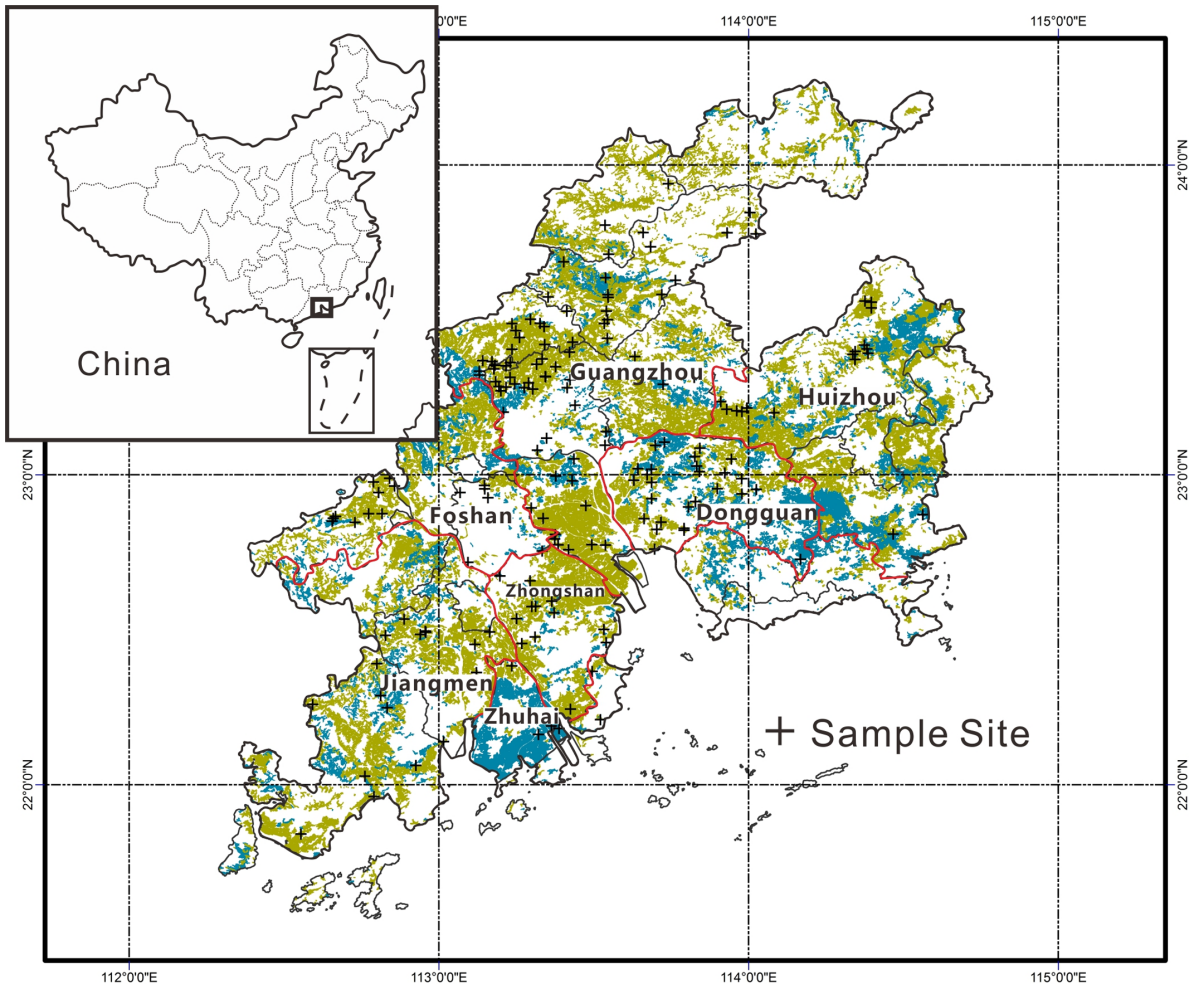
Locations of soil and vegetable samples in the Pearl River Delta area, Guangdong, China. (The yellow and blue areas are the dry lands and the paddy fields, respectively; the red line is the boundary of the city).

The PRD area is located in the south of Guangdong province occupying 41698 km^2^ of land area (Figure 1). The area has a subtropical – tropical monsoon climate with an average annual temperature of 21–22°C and average annual rainfall of 1600–2000 mm. The main soil types in the PRD area are Ultisol, mostly developed on the granite parent materials in the local hills, and paddy soils developed on the fluvial sediments. In the study area, 10–15 crops of vegetables are planted annually, and the rotation of vegetable and rice is applied in mostly paddy soils.

Over the last 30 yr, rapid urbanization and industrialization has taken place in this area. Heavy metal contents in soils and sediments were elevated compared with historical monitoring results [29]. As a result, heavy metal accumulation in agricultural soils has also become increasingly serious in this area because of increasing reliance on fertilizers and agrochemicals [30].

### Field sampling and preparation

Both surface soil samples and vegetable samples used in this study were collected from locations shown in Figure 1. All sample sites were far away at least 100 m from the obviously polluted area such as industries, feedlot, wastewater and highway for avoiding the directly anthropogenic influence. The planting area of each site was larger than 1000 m^2^ for.a certain vegetable species. In this study the vegetable species included Pakchoi (*Brassica rapa chinensis*) (n = 31), Chinese flowering cabbage (*Brassica campestris L. ssp. chinensis*) (n = 37), Leaf mustard (*Brassica juncea Coss*) (n = 16), Romaine lettuce (*Lactuca sativa L. var. longifolia*) (n = 31), Chinese lettuce (*Lactuca sativa L. var. asparagina*) (n = 15), Cauliflower (*Brassica oleracea L.var. botrytis L.*) (n = 7), Water spinach (*Ipomaea aquatic Forssk*) (n = 7), Celery (*Apium graveolens*) (n = 6), Chinese chives (*Allium tuberosum*) (n = 5), Spinach (*Spinacia oleracea*) (n = 5), Amaranth (*Amaranthus mangostanus L.*) (n = 5), watercress (*Nasturtium officinale*) (n = 5).

Vegetables and field soils at a soil depth of 0–15 cm which was the root concentrated layer in this study were collected when the vegetable were suitable for harvest. Each vegetable and soil sample consisted of five subsamples, and combined and mixed well. The fresh vegetable samples were put in clean plastic bags and immediately transported to the laboratory for sample treatment.

The vegetable samples were cleaned with tap water and Milli-Q water, and then the edible parts (including leaves and steams) were separated, weighed (Fresh weight). The washed samples were then dried in an oven at 60°C, and their dry weights (DW) were recorded. The dry vegetable samples were ground to pass through a 250 µm sieve in a steel grinder. Soil samples were air-dried at room temperature (25°C) and ground to pass through a 2 mm nylon sieve. The fine vegetable and soil sample powders were stored in polythene zip bags.

### Chemical analyses

The sieved soil samples were ground further to pass through a 150 µm nylon sieve. The prepared soil samples were digested to dryness using an acid mixture of 10 ml HF, 5 ml HClO_4_, 2.5 ml HCl, and 2.5 ml HNO_3_. Total Cd concentrations in soils were determined by Inductively Coupled Plasma-Atomic Emission Spectrometry (ICP-AES) (Model PS 1000 AT, USA). The recovered soil Cd concentration of the National Research Center for GeoAnalysis soil standard reference materials (SRM) (ESS-4, Beijing, China; Standard value: 0.083 mg kg^−1^) was 0.081 mg kg^−1^ (n = 32). The analytic precision was 2.4% for soil Cd.

Seventy five soil samples were analyzed for exchangeable Cd using the first step of Tessier sequential extraction[31]. The extraction procedure is 2.00 g of air-dried soil (<2 mm) were mixed with 16 ml 1 M MgCl_2_ solution (pH = 7.0), the mixture was shaken for one hour on 25°C. the suspension was filtered immediately after shaking. The Cd concentration in the filtrate solutions were measured by Inductively Coupled Plasma-Atomic Emission Spectrometry (ICP-AES) (Model PS 1000 AT, USA).

The ground vegetable samples were ashed in a muffle furnace for 16 h at 500°C, dissolved in 0.5 M HNO_3_, and diluted to 25 mL with deionized water. Cd concentrations in vegetables were determined by Inductively Coupled Plasma-Atomic Emission Spectrometry (ICP-AES) (Model PS 1000 AT, USA). A plant standard reference material (GSV-4, Beijing, China; Standard value:0.057 mg kg^−1^) was used in order to control the determination quality. The recovered Cd concentration was 0.059 mg kg^−1^ (n = 10). The analytic precision 3.5% for vegetable Cd. The Cd concentration in vegetable samples was expressed on a fresh weight basis.

The pH of soil was measured by taking 10 g of sample into 25 ml of deionized water [32]. The soil organic matter content was measured using potassium bichromate oxidation process [33].

### Data analysis

The transfer factors (TFs) of Cd from soil to vegetable (edible part) were calculated using the following equation [12], [15]–[17], [20], [34]:
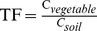
Where *C_vegetable_* is Cd concentration (FW) in the edible parts of vegetables, and *C_soil_* is the total Cd concentration (DW) in the soil where the vegetable was grown.

The data statistical analysis was performed using the Minitab 16 statistical software (Minitab Inc., USA). Spatial interpolation was performed using ordinary kriging. For the low-density sampling, the ordinary kriging estimate can be thought of simply as an optimally weighted average of the data [35]. It provides a best linear unbiased prediction of spatial distribution. The spatial interpolation and contour maps displaying the spatial distribution of Cd concentrations in soils and vegetables, and transfer factors were produced based on the geostatistical analysis by using the software of ArcGIS 9.0.

## Results and Discussions

### Cd concentrations in vegetable soils

**Figure 2:**
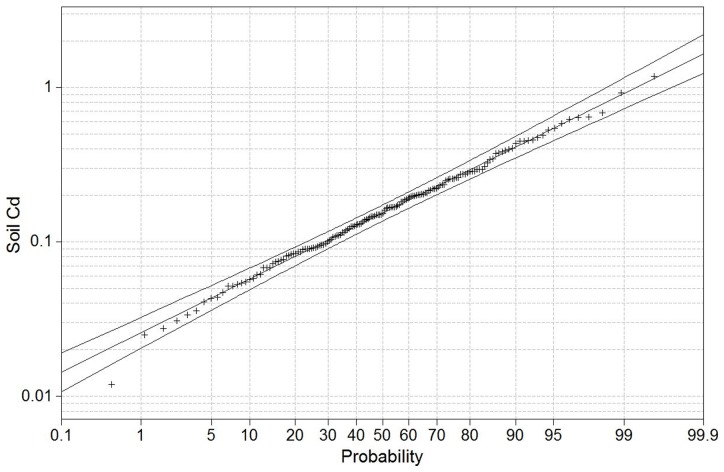
Lognormal probability plot for total Cd concentration in vegetable soils in the Pearl River Delta area, Guangdong, China (mg kg^−1^, DW).

Total Cd concentrations in soils range from 0.012 to 1.18 mg kg^−1^ in the PRD area (Table 1). These values fit a log normal distribution (Figure 2), therefore the geometric mean (GM) value of 0.158 mg kg^−1^ and geometric standard deviations (GSD) of 1.39 were used to represent the central tendency and variations of the data. The present GM value was much higher than its background concentration (0.04 mg kg^−1^) of soil in Guangdong province [35] and significantly lower than Cd mean concentrations of 0.858 mg kg^−1^ in the reclaimed tidal flat soil and 1.4–1.8 mg kg^−1^ in some Cd-contaminated vegetable soils in the PRD area [20], [21]. The present results reflect the overall level of soil Cd concentration on the region al scale, not is responsible for local point-source contamination.

**Table 1:** Descriptive statistics of soil Cd concentrations (mg kg^−1^, DW) and vegetable Cd concentration (mg kg^−1^, FW) in the PRD area.

Sample Site	n	Soil Cd concentration			Vegetable Cd concentration			pH (H_2_O)	OM^e^ (%)
		Mean ± SD	Range	C.V. (%)	Mean ± SD	Range	C.V. (%)	Mean ± SD	Mean ± SD
Dongguan	27	0.138±0.079	0.041–0.433	57.2	0.024±0.012	0.004–0.057	48.4	5.76±0.87	2.58±0.70
Foshan	16	0.291±0.200	0.025–0.685	69.0	0.017±0.011	0.002–0.037	62.5	5.84±1.12	2.61±1.01
Guangzhou	73	0.226±0.190	0.027–1.180	84.3	0.027±0.024	0.002–0.082	86.8	6.34±0.96	2.44±0.66
Jiangmen	17	0.188±0.157	0.012–0.644	83.9	0.014±0.016	0.002–0.068	117.8	5.49±0.67	2.28±0.90
Zhuhai	6	0.204±0.160	0.044–0.474	78.5	0.008±0.005	0.001–0.014	58.1	6.11±0.99	2.34±0.63
Zhongshan	13	0.316±0.150	0.083–0.530	47.3	0.022±0.021	0.001–0.067	92.7	6.02±0.97	2.45±0.90
Huizhou	18	0.107±0.052	0.052–0.200	39.6	0.026±0.018	0.010–0.082	69.1	6.69±0.43	2.41±0.62
Total	170	0.208±0.1690.158 (1.39)^d^	0.012–1.180	81.2	0.024±0.0190.015 (1.57)^d^	0.002–0.082	84.3	6.12±0.96	2.42±0.74
Threshold		0.3^a^			0.05^b^; 0.2^c^				

a The maximum permissible concentrations of Cadmium for agriculture soils (SEPAC, 1995).

b the maximum permissible concentrations of Cadmium for non-environmental pollution vegetable (AQSIQ, 2001).

c The maximum level of Cadmium for leaf vegetables (MOH, 2012).

d Geometric Mean (Geometric standard deviation).

e Orgnic matter.

**Figure 3:**
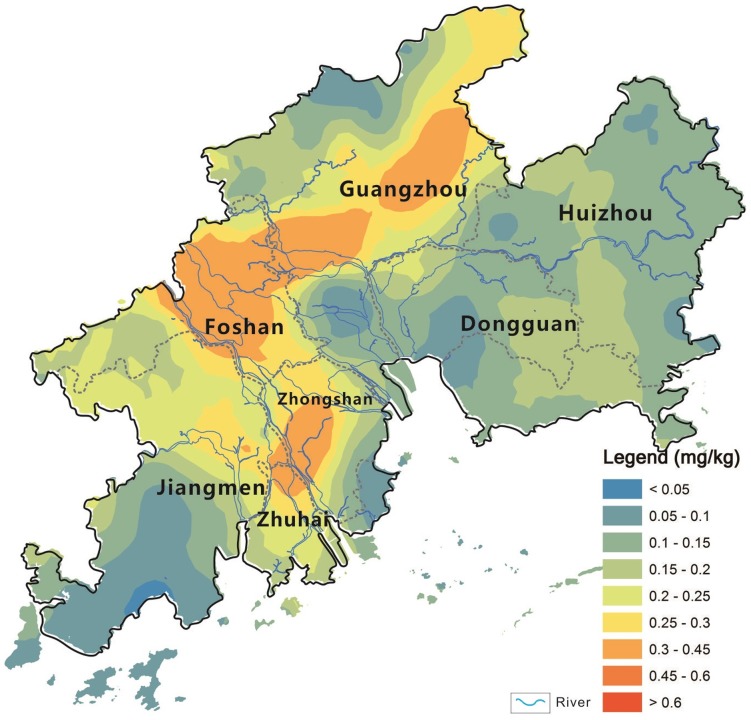
Spatial pattern of total Cd concentration in the soils in the PRD area (mg kg^−1^, DW).

According to the maximum permissible concentrations of cadmium for agriculture soils of 0.3 mg kg^−1^ [8], thirty vegetable soils were contaminated with Cd in the study area, mainly located in the Guangzhou, Zhongshan, and Foshan cities. The soil Cd mean concentration was up to 0.316 mg kg^−1^ in the Zhongshan city, followed by 0.291 mg kg-1 in the Foshan city, 0.226 mg kg-1 in the Guangzhou City, and 0.204 mg kg-1 in the Zhuhai City (Table 1). Generally, spatial characteres of soil Cd show that the western areas (including Foshan, Guangzhou, Zhongshan, Zhuhai) influenced by the West River and North River has the higher Cd concentration than the eastern areas (including Dongguan and Huizhou) influenced by the East River (Figure 3). The results coincided with spatial characters of other heavy metal concentrations (Pb, Zn) in sediments in the PRD area, that is, there were higher heavy metal concentrations in sediments of the West River and North River than that of the East River [36]. The spatial correlation between soil Cd cocnetrtiaons and regional rivers suggested that the cadmium produced by the mining and smelting activities in the upper reaches of the West River and North River was the main source of Cd in soils of the PRD area, and the West River and North River was the important transfer approach for Cd entering into surrounding soils through river irrigation.

**Figure 4:**
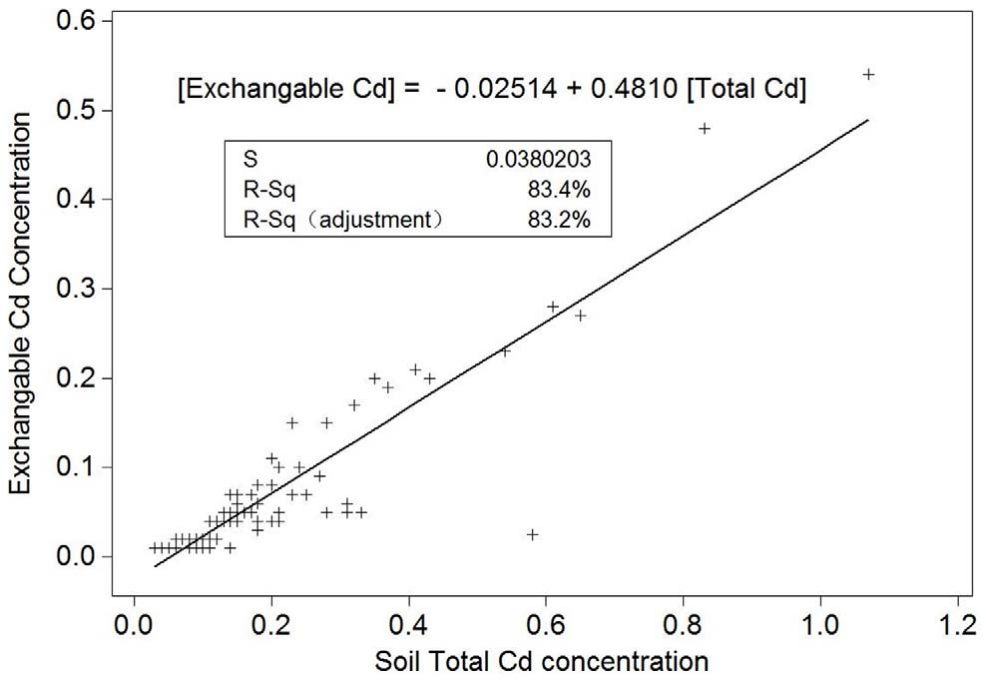
Relationship between exchangeable Cd concentration and total Cd concentration in vegetable soils (mg kg^−1^, DW).

Although many literatures pointed out that metal availability in soil is of main concern, because the available concentration is an indication of the amount available for plant uptake, and provided some methods to determine plant availability of heavy metals [12], [21], [24]. Our results reveal that the significant positive correlation between soil total Cd and exchangeable Cd was shown in Figure 4, which can be described by a linear equation:
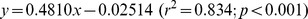
Where y is the soil exchangeable Cd concentration, *x* refers to soil total Cd concentration.

This indicated that exchangeable Cd concentration in soils in the PRD area was mainly controlled by the soil total Cd concentration.

### Cd concentrations in vegetables

The present results show that in the PRD area the range of Cd concentration in all vegetable samples was 0.002–0.082 mg kg^−1^ with a geometric mean value of 0.015 mg kg^−1^ (Table 1). In this study Cd concentrations in all vegetable samples met the national standard of Safe vegetables (0.2 mg kg^−1^), 150 vegetable samples met the standard of No-Polluted vegetables (0.05 mg kg^−1^). Twenty vegetable samples were in between Safe vegetables and No-polluted vegetables, of which14 vegetable sample sites were in the Guangzhou.

**Figure 5:**
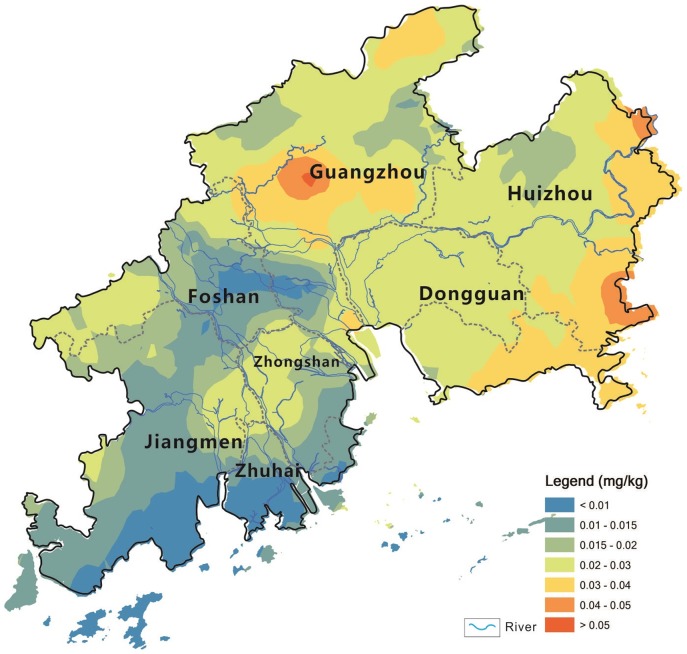
Spatial pattern of Cd concentrations in the vegetables in the PRD area (mg kg^−1^, FW).

According to the different cities, the mean vegetable Cd concentration, was in the order of Guangzhou (0.027 mg kg^−1^)> Huizhou (0.026 mg kg^−1^)> Dongguan (0.024 mg kg^−1^)> Zhongshan (0.022 mg kg^−1^)> Foshan (0.017 mg kg^−1^)> Jiangmen (0.014 mg kg^−1^)> Zhuhai (0.008 mg kg^−1^) (Table 1). This order is significantly different from Cd concentrations in soils. The spatial pattern of Cd concentrations in vegetables showed that no spatial correlation with that of total Cd cocentrtaions in soils on the regional scale (Figure 3 and Figure 5).

The mean and range of Cd concentrations in various vegetable species were listed in Table 2. In the study area the dominant vegetable species are the pakchoi, Chinese flowering cabbage, leaf mustard, Romaine lettuce and Chinese lettuce for the local residents. The mean Cd concentrations in these vegetables range from 0.022 mg kg^−1^ to 0.031 mg kg^−1^ (Table 2). The amaranth vegetables had the highest Cd concentration of 0.082 mg kg^−1^ with mean value of 0.06 mg kg^−1^. This value was closed to 0.078 mg kg^−1^ in amaranth on the reclaimed tidal flat soil reported by Li et al [20]. Compared with the previous researches on the Cd-contaminated soil [20], [21], the present results revealed that there were low Cd concentrations of various vegetables in the study area.

**Table 2:** Cd concentration (mg kg^−1^, FW) in different vegetable species and the TF values in the PRD area.

Vegetable species	n	Cd		TF	
		Mean ± SD	Range	Mean ± SD	Range
Pakchoi (*Brassica rapa chinensis*)	31	0.023±0.015	0.002–0.074	0.222±0.220	0.010–0.920
Chinese flowering cabbage (*Brassica campestris L. ssp. chinensis*)	37	0.022±0.015	0.001–0.068	0.160±0.142	0.025–0.774
Leaf mustard (*Brassica juncea Coss*)	16	0.023±0.014	0.001–0.054	0.532±1.087	0.022–4.50
Romaine lettuce (*Lactuca sativa L. var. longifolia*)	31	0.026±0.021	0.003–0.082	0.148±0.111	0.001–0.371
Chinese lettuce (*Lactuca sativa L. var. asparagina*)	15	0.031±0.026	0.005–0.078	0.188±0.219	0.017–0.821
Cauliflower (*Brassica oleracea L.var. botrytis L.*)	7	0.010±0.013	0.002–0.032	0.048±0.042	0.014–0.103
Water spinach (*Ipomaea aquatic Forssk*)	7	0.011±0.019	0.001–0.053	0.045±0.052	0.003–0.117
Celery (*Apium graveolens*)	6	0.043±0.020	0.019–0.064	0.234±0.115	0.110–0.376
Chinese chives (*Allium tuberosum*)	5	0.027±0.032	0.001–0.074	0.229±0.241	0.001–0.563
Spinach (*Spinacia oleracea*)	5	0.037±0.027	0.006–0.067	0.213±0.034	0.182–0.262
Amaranth (*Amaranthus mangostanus L.*)	5	0.060±0.017	0.030–0.082	0.753±0.442	0.258–1.577
watercress (*Nasturtium officinale*)	5	0.021±0.020	0.002–0.039	0.087±0.046	0.003–0.129
Total	170	0.024±0.019	0.001–0.082	0.189±0.211	0.001–4.50

### Relationships between Cd in soils and in vegetables

Many studies have shown that the concentration of heavy metals in vegetables is influenced by many factors, such as total concentration of heavy metals in soils, vegetable species, soil pH value, soil organic matter, climate, atmospheric depositions and temperature [19], [21], [37]–[39]. McBride [40] suggested that combination of soil pH and soil total Cd concentration was reasonably predictive of Cd concentration in the above-ground plant tissue.

**Figure 6:**
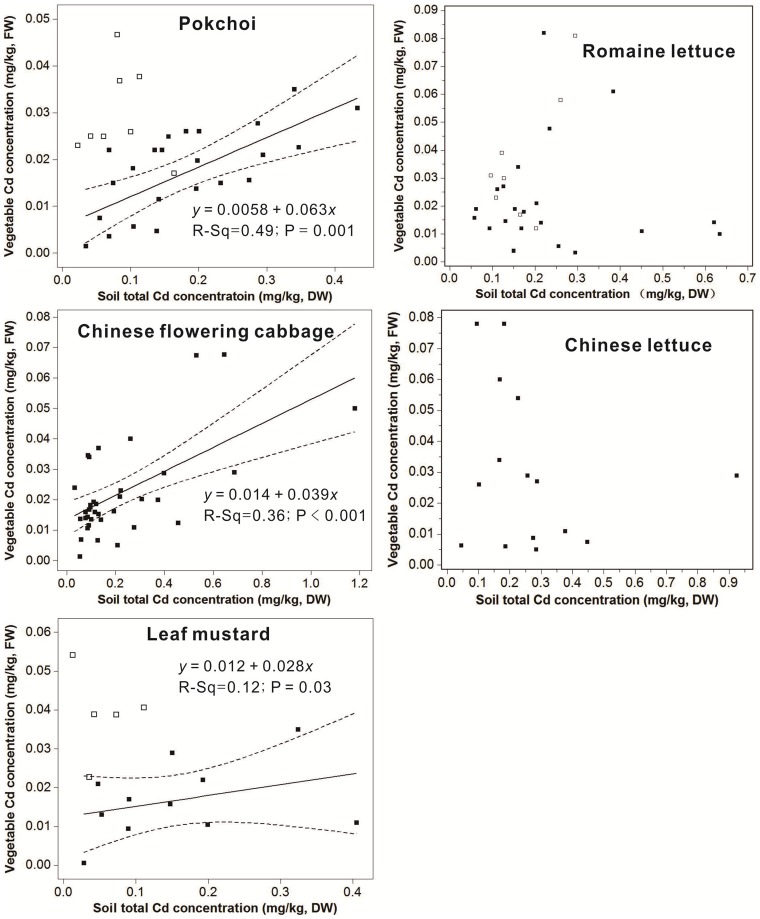
Relationships between soil total Cd concentrations (mg kg^−1^, DW) and vegetable Cd concentrations (mg kg^−1^, FW) for five species of main vegetables (soil pH: 3.79–7.72) (hollow diamonds represented samples with lower pH (3.79−5.00) removed for the regression analysis; the solid line is the sample regression line; dotted lines indicated the 95% confidence interval).

In this study, we discussed the correlation between Cd concentrations in five species of dominant leaf vegetables and Cd concentrations in soils. The results revealed that in the condition of soil pH>5, Cd concentrations in both pakchoi and Chinese flowering vegetables had obvious positive correlations with Cd concentrations in soils; and Cd concentrations in leaf mustard vegetables and Cd concentrations in soils had a weak positive correlation (Figure 6). These can be described by linear equations, respectively:
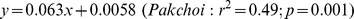


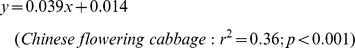


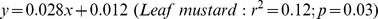
Where *y* is the Cd concentration in the various vegetable, *x* is the soil total Cd concentration.

The results of the line regression indicated that in the condition of soil pH>5, soil total Cd concentrations explained 49%, 36% and 12% of the variability of Cd concentration in Pakchoi and Chinese flowering cabbage and leaf mustard, respectively. But Cd concentrations in the Romaine lettuce and Chinese lettuce had no correlations with total Cd concentrations in soils (Figure 6). This phenomenon should be related with various vegetable families. Pakchoi, Chinese flowering cabbage and leaf mustard belong to the brassica of cruciferous plants; Romaine lettuce and Chinese lettuce belong to the Lactuca of Compositae plants. The difference of correlations between Cd concentrations in vegetables and total Cd concentrations in soils should be the result of different absorption capacity of various vegetable families to Cd. And the result showed that the influence of soil total Cd concentrations on the Cd in various vegetable species is obviously different [39], [41].

When soils pH is less than 5, total Cd concentrations in soils were low because the environmental capacity of soil Cd was small in acid soils due to the strong eluviation, but Cd concentrations in the pakchoi and leaf mustard vegetables were much higher than the reference line (Figure 6). Obviously, low soil pH can raise the available Cd content for pakchoi and leaf mustard vegetables even in the soils with low Cd concentrations.

**Figure 7:**
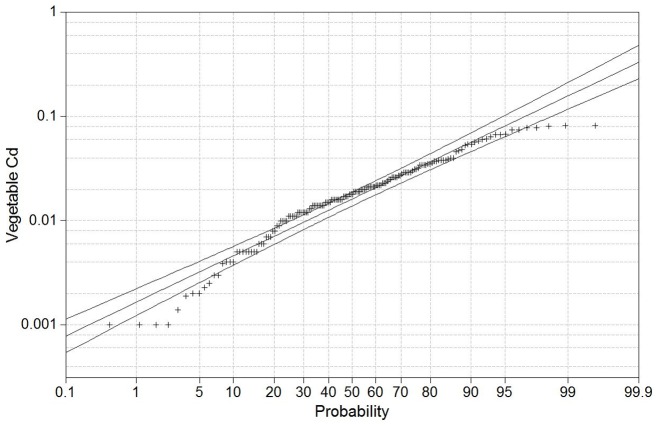
Lognormal probability plot for vegetable Cd concentrations in the Pearl River Delta area, Guangdong, China (mg kg^−1^, FW).

The analysis of Pearson correlations among soil Cd, vegetable Cd, soil pH, organic matter and transfer factor showed that there was no obvious correlation among soil Cd, vegetable Cd, organic matter and soil pH; significant correlations were be found between transfer factor and soil Cd (r^2^ = −0.295) and between transfer factor and vegetable Cd (r^2^ = 0.435) (Table 3). Our results confirmed that soil pH played important role to Cd absorption of vegetables, but there was no linear correlation between soil pH and Cd concentration in vegetables in this study. Moreover, The lognormal probability distribution graph shows that some vegetable samples of upper tail had less high value than would be expected (Figure 7), suggesting the vegetable's ability to absorb Cd would be decreasing with the increasing the Cd content in the vegetables.

**Table 3:** Pearson correlations of soil Cd, vegetable Cd, soil pH, organic matter and Transfer factor.

	soil Cd	vegetable Cd	pH	Organic matter
vegetable Cd	0.205(0.088)			
pH	0.033(0.784)	0.137(0.258)		
Organic matter	0.065(0.590)	−0.007(0.952)	0.076(0.532)	
TF	−0.295(0.013)	0.435(0.000)	−0.143(0.239)	−0.103(0.396)

Cell content: Pearson correlation (p-value).

### Transfer factors of Cd from soil to vegetable

The soil-to-vegetable transfer factors (TFs) reflected the ability of vegetables to take up soil metals. TFs varied significantly with plant species [12], [19], [21], [38], [39], and were commonly viewed as a “constant” for a given plant species and a given metal. TFs were used as an important character index for establishing the soil environmental quality criteria or assessing the health risk of soil contamination.

**Figure 8:**
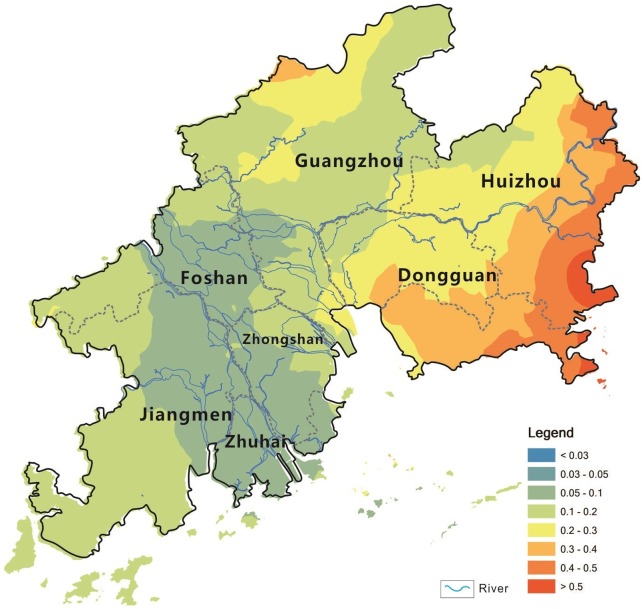
Spatial pattern of soil–to–vegetable transfer factor of Cd in the PRD area.

In this study, the TFs of Cd from soil to vegetable for the 12 vegetable species varied from 0.045 in Water spinach to 0.753 in Amaranth (Table 2). The TF mean values in the main five species of leaf vegetables in the study area were 0.532 for leaf mustard, 0.222 for Pakchoi, 0.188 for Chinese lettuce, 0.160 for Chinese flowering cabbage, and 0.148 for Romaine lettuce. Compared with TFs of Cd reported by Wang et al [12], and Hu et al [21], these values were much higher because of the lower Cd concentration in soils. The spatial pattern of TFs also showed that the sample sites with high TFs were always corresponding to the lower Cd concentrations in soils in the study area (Figure 3 and Figure 8).

The regression equations between the TF values and total Cd concentrations in soils can be described by power model (y  =  ax^b^) for five kinds of main vegetables:
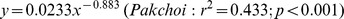


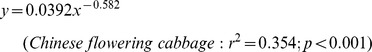


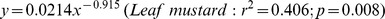


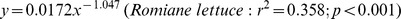


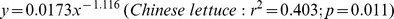
Where y is the soil-to-vegetable transfer factor of Cd, x is the total Cd concentration in the soils.

**Figure 9:**
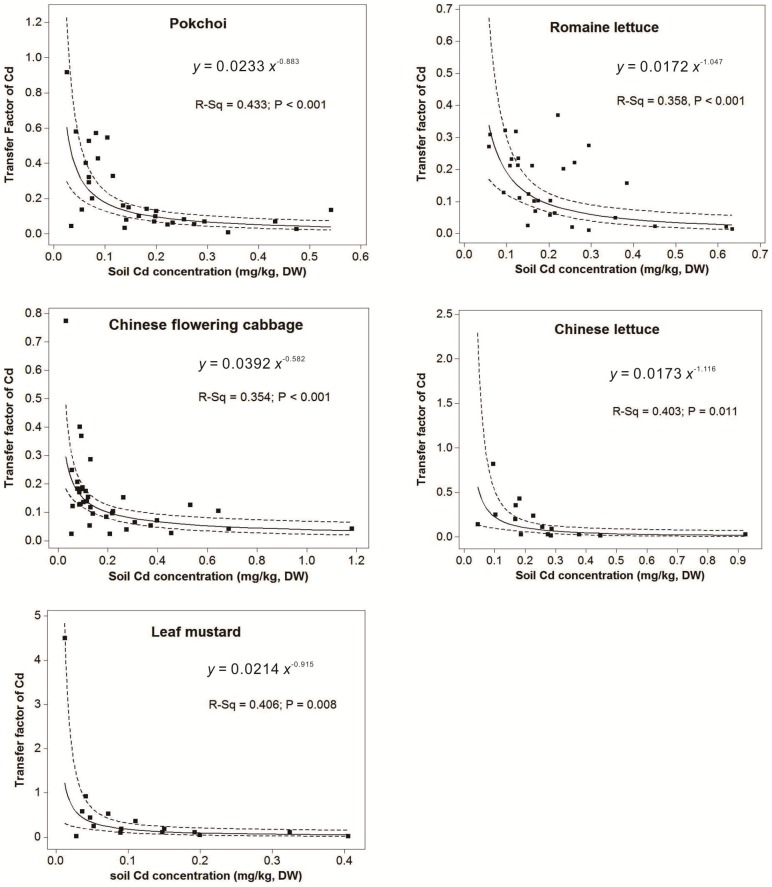
Relationships between soil-to-vegetable transfer factors of Cd and total Cd concentrations in soils for five species of main vegetables (the solid line is the sample regression curve; dotted lines indicated the 95% confidence interval).

For main leaf vegetables, the transfer factors of Cd decreased with increasing total Cd concentrations (Figure 9), suggesting that the ability of vegetable to take up soil Cd decreased with the total soil Cd increasing. Figure 9 also illustrated that when soil Cd concentration was low (about 0.1–0.2 mg kg^−1^), the TFs decreased sharply; with the gradual increasing of soil total Cd concentration, TFs decreased only slowly, till to a stable value. Moreover, the 95% confidence interval of regression lines showed that there were a larger error range at the low Cd concentration interval of soils (0–0.1 or 0.2 mg kg^−1^) for the various leaf vegetable species, and when soil Cd concentrations were greater than 0.1 or 0.2 mg kg^−1^, the TFs of various vegetables would be reasonable with a lower error range for the assessment of environmental risk (Figure 9). As a result, for carrying out rational environmental health assessment in different Cd concentrations of soils, the present results indicated that the fit line of TFs for vegetable species should be discussed to divide into two parts, including the sharply decrease part and the slowly decrease part, according to the gradual increasing of soil total Cd concentration. This work is agree with the suggestion proposed by Wang et al [12] that the transfer factors of a given crop-metal system estimated from the regression model between the TF values and the corresponding soil metal concentration, are much more reasonable than using the arithmetic means or geometric means at a given soil metal concentration.

## Conclusions

The present results showed that the total Cd concentration in soils is safe for planting leaf vegetables in the study area. All vegetable samples met the national standard of safe vegetables, and 88% of which met the standard of no-polluted vegetables. Total Cd concentrations in soils and soil pH played an important role to Cd absorption of vegetables, but there were no obviously linear correlations. There was no spatial correlation between total Cd concentrations in soils and Cd concentrations in vegetables. For a givenl vegetable species the regression model between the TF value and the corresponding total Cd concentrations in soils could be used to estimate the transfer abilities of Cd from soil to vegetable at a given total Cd concentration in soils.
